# Patient-Derived Exosomes as siRNA Carriers in Ovarian Cancer Treatment

**DOI:** 10.3390/cancers16081482

**Published:** 2024-04-12

**Authors:** Aasa Shimizu, Kenjiro Sawada, Masaki Kobayashi, Yukako Oi, Tadashi Oride, Yasuto Kinose, Michiko Kodama, Kae Hashimoto, Tadashi Kimura

**Affiliations:** 1 Department of Obstetrics and Gynecology, Osaka University Graduate School of Medicine, Suita 565-0871, Japan; aasashimizu@gyne.med.osaka-u.ac.jp (A.S.); macha_111@gyne.med.osaka-u.ac.jp (M.K.); yukakooi.08114@gyne.med.osaka-u.ac.jp (Y.O.); t.oride@gyne.med.osaka-u.ac.jp (T.O.); kinose0205@gyne.med.osaka-u-ac.jp (Y.K.); mkodama@gyne.med.osaka-u.ac.jp (M.K.); kae.h@gyne.med.osaka-u.ac.jp (K.H.); tadashi@gyne.med.osaka-u.ac.jp (T.K.); 2 Department of Obstetrics and Gynecology, The University of Chicago, Chicago, IL 60637, USA

**Keywords:** siRNAs, exosomes, ovarian cancer, omentum, fibroblasts, peritoneal dissemination, *MET*

## Abstract

**Simple Summary:**

We demonstrated that small interfering RNA replacement therapy using engineered exosomes exhibits promising effects in treating the peritoneal dissemination of ovarian cancer. Considering that patients with ovarian cancer routinely undergo omentectomy and that exosomes can be extracted from omental fibroblasts without difficulty, engineered exosomes can be used as siRNA delivery carriers for future molecular targeted therapies, ultimately resulting in the personalized treatment of patients with ovarian cancer.

**Abstract:**

RNA interference is a powerful gene-silencing tool with potential clinical applications. However, its therapeutic use is challenging because suitable carriers are unavailable. Exosomes are stable small endogenous vesicles that can transport functional molecules to target cells, making them ideal small interfering RNA (siRNA) carriers. Herein, we elucidated the therapeutic potential of patient-derived exosomes as an siRNA carrier for ovarian cancer (OC) treatment. The exosomes were extracted from the culture medium of primary fibroblasts collected from the omentum of patients with OC during surgery. MET proto-oncogene, receptor tyrosine kinase (*MET*) was selected for gene silencing, c-Met siRNAs were synthesized and loaded into the exosomes (Met-siExosomes) via electroporation, and the treatment effect of the Met-siExosomes was assessed in vitro and in vivo. The Met-siExosomes downregulated the c-Met protein levels and inhibited OC cell proliferation, migration, and invasion. In xenograft experiments using SKOV3-13 and ES-2 cells, Met-siExosomes were selectively extracted from peritoneally disseminated tumors. Intraperitoneal treatment suppressed the c-Met downstream targets in cancer cells and prolonged mouse survival. The synthesized siRNAs were successfully and selectively delivered via the exosomes to intraperitoneally disseminated tumors. As patients with OC routinely undergo omentectomy and abundant fibroblasts can be easily collected from the omentum, patient-derived exosomes may represent a promising therapeutic siRNA carrier to treat OC.

## 1. Introduction

Ovarian cancer (OC) is one of the deadliest cancers in women and the fifth leading reason for cancer-related mortality among women [1]. More than two-thirds of patients with OC are diagnosed at advanced stages and present extensive peritoneal dissemination with massive ascites, resulting in a poor prognosis [2]. With the approval of drugs such as bevacizumab and poly (ADP-ribose) polymerase (PARP) inhibitors, the treatment strategies for OC have dramatically changed [3,4]. However, the prognosis of patients with OC has not significantly improved, which is primarily because of the difficulty in treating its peritoneal dissemination. Therefore, novel strategies for overcoming this fatal disease that focus on the peritoneal dissemination are warranted.

In addition to conventional therapies, the use of small interfering RNA (siRNA) in clinical settings has been identified as a future therapeutic option for cancer treatment since 2000 [5]. Theoretically, siRNAs can silence disease-related genes in a sequence-specific manner. However, despite the two-decades-long journey since their discovery, the clinical application of siRNAs against cancer remains challenging because they are unstable in body fluids and toxic to nontumor cells [6]. Therefore, a novel siRNA delivery system is required to use these molecules in clinical settings. To address these problems, exosomes, which are naturally present in the human body, have garnered attention as promising novel siRNA delivery systems.

Exosomes are nanosized vesicles released by a type of cells that are involved in the intercellular exchange of DNA, RNA, proteins, and other cellular components within the lipid bilayer. Extracellular vesicles can be categorized depending on their source: exosomes, microvesicles, and apoptotic bodies. Exosomes, with diameters spanning 30–150 nm, are produced inside multivesicular endosomes, merge with the plasma membrane, and are subsequently discharged, serving as intermediaries in the endosomal pathway [7]. Exosomes contribute to cell–cell communication, are stable in body fluids, can evade the immune system, and have a natural targeting capability [8]. Therefore, the potential of exosomes has renewed our interest in their use as delivery systems for various therapeutics [9,10,11]. Regarding OC, recent reviews revealed that exosomes are involved in remodeling the tumor microenvironment, promoting tumor angiogenesis and metastasis, regulating immune metastasis, and the acquisition of chemotherapy resistance [12,13]. In addition, if patient-derived exosomes can be used as transport carriers, immune reactions during administration may be avoided. Various preclinical studies have revealed the potential of exosomes as carriers for nucleic acid compounds (such as siRNA and shRNA), proteins (such as 20S proteasome and catalase), and drugs (such as dopamine, paclitaxel, and doxorubicin) [11,14,15]. Furthermore, we recently demonstrated the efficacy of exosomes as microRNA carriers [16,17].

The key to the effective clinical application of exosomes for the transduction of siRNA into relapsed tumors is the collection of sufficient amounts of patient-derived exosomes. Most patients with OC undergo surgery, including partial omentectomy, as the primary treatment modality. As the omentum contains fibroblasts which secrete abundant exosomes [16], we attempted to use primary fibroblasts from the omentum as an exosome source for delivering siRNAs.

In this study, we evaluated the potential use of exosomes as siRNA carriers, investigated whether siRNA replacement therapy could be performed with patient-derived exosomes from the omentum, and developed a novel precision therapy for OC.

## 2. Materials and Methods

### 2.1. Materials

Dulbecco’s modified Eagle medium (DMEM; #08458-45) and RPMI-1640 were obtained from Nacalai Tesque (Kyoto, Japan). Fetal bovine serum (FBS; #172012) and hepatocyte growth factor (HGF; #H1404) were procured from Sigma-Aldrich (St. Louis, MO, USA). Antibodies against c-Met (C-28; #161), E-cadherin (#8426), and cytokeratin-18 (#6259) were purchased from Santa Cruz Biotechnology (Dallas, TX, USA). Antibodies against CD9 (EXOAB-CD9A-1), CD63 (EXOAB-CD63A-1), CD81 (EXOAB-CD81A-1), and α-SMA (#14395-1-AP) were obtained from Proteintech (Rosemont, IL, USA). Antibodies against β-actin (#4967), phospho-AKT (Ser473; #9271), extracellular signal-regulated kinase (ERK) 1/2 (3A7; #9107), phospho-ERK 1/2 (p-ERK 1/2; Thr202/Tyr204, E10; #9106), and AKT (#9272) were purchased from Cell Signaling Technology (Danvers, MA, USA). Lipofectamine 3000 (#L3000008), TRIzol (#15596-018), and anti-phospho-c-Met antibody (pypypy1230/1234/1235; #44888G) were obtained from Life Technologies (Carlsbad, CA, USA). The antibody against vimentin (#M0725) was procured from DAKO (Glostrup, Denmark). Streptavidin 10 nm gold (#AC-10-04-05) was purchased from Cosmo Bio (Tokyo, Japan). 

### 2.2. Cell Culture

BJ cells, fibroblasts established from the human skin, and the CAOV3, ES-2, and SKOV3 cell lines were procured from the American Type Culture Collection (Manassas, VA, USA). SKOV3 cells stably expressing the beta subunit of human gonadotropin and firefly luciferase were generated as described previously and named SKOV3-13 [18]. The cells were cultured in DMEM supplemented with 10% FBS and incubated at 37 °C in 95% O_2_ and 5% CO_2_. The cells were authenticated via short tandem repeat DNA profiling at Takara-Bio Inc. (Kusatsu, Japan) and were used within 6 months of resuscitation. Fibroblasts were primarily cultured from the normal omentum of patients undergoing gynecological surgery at the Osaka University Hospital [19]. All the patients provided written informed consent before surgery.

### 2.3. Immunohistochemistry

Fibroblasts were plated on chamber slides, fixed with 4% paraformaldehyde, and stained with primary antibodies against cytokeratin-18 (1:50), propyl 4-hydroxylase (1:1000), or vimentin (1:50) for 1 h. Tumor samples were fixed with 10% neutral-buffered formalin, embedded in paraffin, and sectioned. The primary antibodies were as follows: c-Met antibody (1:400, #51067; Abcam), p-ERK 1/2 antibody (1:200, #9106; Cell Signaling), and integrin β1 antibody (1:200, #610467; BD Biosciences. A BX53 microscope (Olympus, Tokyo, Japan) was used to capture optical images.

### 2.4. Public Database Analysis

*MET* expression (Ensemble ID: ENSG00000105976.14) in OC was evaluated using Gene Expression Profiling Interactive Analysis (GEPIA), which includes The Cancer Genome Atlas and Genotype-Tissue Expression (http://gepia.cancer-pku.cn/detail.php?gene=MET, accessed on 14 March 2023). The Kaplan–Meier plotter, which includes 1,435 patients with OC, was used to determine the prognostic value of *MET* expression in OC, as previously described [16]. Patients were categorized into two groups based on the lower-tertile threshold using the ABHD17B (226016_at) probe: “high”- and “low”-*MET*-expression groups. Then, a Kaplan–Meier survival plot was used to compare the two patient groups.

### 2.5. Fibroblast Purification from Omental Tissue

Specimens of human omentum were collected from patients undergoing surgery for stage I borderline ovarian tumors or stage I ovarian cancers. Informed consent was obtained from all the patients before surgery. Omentum without gross abnormal appearances was used for the subsequent experiments. The collected omentum was minced into 2–3 cm^2^ pieces and incubated in a shaker with 10 mL of PBS and 10 mL of 0.25% trypsin/25 mM EDTA at 37 °C for 30 min. The solution was aspirated to remove the mesothelial cells. To isolate the fibroblasts, the tissue was further digested with 100 units of hyaluronidase and 1000 units of collagenase type 3 in 100 mL PBS at 37 °C for 6 h while shaking. The tissue was discarded and the solution containing the cells in a suspension was centrifuged at 1500 rpm for 5 min. The supernatant was aspirated to remove the adipocyte [20], and the pellet was washed twice with PBS. The fibroblasts were seeded with RPMI 1640 supplemented with 20% FBS, 100 U/mL penicillin, and 100 μg/mL streptomycin [21].

### 2.6. Isolation of Exosomes

A differential centrifugation method described previously was used to isolate the exosomes from the cell culture medium [16,19]. Briefly, a conditioned medium containing exosome-depleted FBS (prepared via ultracentrifugation at 100,000× *g* at 4 °C for 18 h) was prepared by incubating the cells grown at sub-confluence for 48 h. Subsequently, the collected supernatant was sequentially centrifuged at 300× *g* for 10 min, 2000× *g* for 10 min, and 10,000× *g* for 30 min. The supernatant was filtered using 0.2 µm filters and then ultracentrifuged at 100,000× *g* using the SW 32Ti rotor (Beckman Coulter, Brea, CA, USA) for 84 min. The supernatant was discarded, and the pellet was resuspended in PBS and ultracentrifuged at 100,000× *g* for 84 min. Finally, the pellet containing the purified exosomes was resuspended in PBS and used for a subsequent analysis. The Lowry method (Bio-Rad, Hercules, CA, USA) was used to measure the content of exosomal proteins.

### 2.7. Synthesis of siRNAs against MET

Two sets of FAM-labeled predesigned siRNA duplexes directed against human *MET* (NM_004580.3-735slcl) were synthesized by Japan Bioservice (Saitama, Japan). The sequences of siRNA-3270 and siRNA-404 were as follows: 5′-GUGCAGUAUCCUCUGACAGTT-3′ (sense) and 5′-CUGUCAGAGGAUACUGCACTT-3′ (antisense) and 5′-CUACAUUUAUGUUUUAAAUTT-3′ (sense) and 5′-AUUUAAAACAUAAAUGUAGTT -3′ (antisense), respectively. Scrambled siRNA [5′-UUCUCCGAACGUGUCACGUTT-3′ (sense) and 5′-ACGUGACACGUUCGGAGAATT -3′ (antisense)] was used as the negative control. 

### 2.8. Electroporation of the siRNAs into Exosomes

The Gene Pulser II system (Bio-Rad Laboratories) was used to electroporate the siRNAs into fibroblast-derived exosomes. Approximately 10^9^ exosomes, measured using qNano (Meiwaforsis, Tokyo, Japan), and 1 μg of the synthesized siRNA were mixed with 400 μL of electroporation buffer (1.15 mM potassium phosphate, pH 7.2; 25 mM potassium chloride; and 21% Optiprep). Then, the mixture was electroplated using a single 4 mm cuvette with the Gene Pulser X cell Electroporation System (#165-2081; Bio-Rad). Thereafter, 400 μL of the RNAi exosome mixture was added to the cuvette, followed by electroporation at 400 V, 125 μF, and ∞ ohms. The cuvette was immediately transferred to ice. The resultant mixture was called Met-siExosomes and used in the subsequent experiments.

### 2.9. Western Blotting

A total of 1 × 10^5^ cells were plated in a six-well plate and lysed with a cell lysis buffer (#9803; Cell Signaling Technology). The cell lysates (15 µg) were subjected to sodium dodecyl sulfate–polyacrylamide gel electrophoresis using 5–20% SuperSep Ace (#197-15011; Wako Pure Chemical Industries, Osaka, Japan) and transferred onto polyvinylidene difluoride membranes (#10600029; GE Healthcare, Little Chalfont, UK). Subsequently, the membranes were incubated with primary antibodies against Met (1:1000), phosphor Met (1:1000), AKT (1:1000), p-AKT (1:1000), ERK (1:1000), p-ERK (1:1000), CD63 (1:500), CD81 (1:500), CD9 (1:500), and β-actin (1:1000). Then, they were incubated with a corresponding horseradish peroxidase-conjugated secondary IgG. An electrochemiluminescence system (PerkinElmer, Waltham, MA, USA) was used to visualize the proteins.

### 2.10. Electron Microscopy

Electron microscopy was performed as previously described [16]. A transmission electron microscope (H-7650; Hitachi, Ltd., Tokyo, Japan) was used for the observations.

### 2.11. Cell Proliferation Assay

OC cells were plated onto 96-well plates at a density of 5 × 10^3^ cells/well in 10% FBS/DMEM for 24 h. They were then incubated with 0.1% bovine serum albumin (BSA)/DMEM. The proliferation of the cells was measured using Life Technologies’ CyQUANT^®^ Cell Proliferation Assay Kit (C7026). 

### 2.12. Matrigel Invasion Assay

The OC cells were treated with Met-siExosomes or the corresponding control exosomes incorporated with scrambled siRNA (control siExosomes) and plated on a modified Boyden chamber system coated with 25 µg of Matrigel in DMEM with 0.1% BSA. The cells were allowed to invade the lower chamber containing 10% FBS/DMEM for 24 h, and a cotton swab was used to remove the non-invading cells. Subsequently, the invading cells on the underside of the filter were counted. 

### 2.13. Wound-Healing Assay

The OC cells were plated on a six-well plate and treated with Met-siExosomes or control exosomes for 24 h. A 200 µL micropipette was used to scratch the wounds, followed by thoroughly washing the cultures with PBS to remove the detached cells. The cell cultures were incubated with 1% FBS/DMEM for 24 h. The width of each scratch was measured in triplicate.

### 2.14. MET Knockdown in OC Cells

*MET* expression was transiently knocked down in the SKOV3, CAOV3, and ES-2 cells using a predesigned siRNA duplex directed against *MET* at a concentration of 200 nmol/L and Lipofectamine 3000.

### 2.15. RT-qPCR Analysis 

The StepOnePlus Real-Time PCR System (Applied Biosystems, Foster City, CA, USA) was used for RT-qPCR. The TRIzol reagent was used to extract the total RNA, which was transcribed into cDNA using the TaqMan RNA Reverse Transcription Kit (#4366596; Applied Biosystems). A TaqMan assay (*MET*; Hs01565584_m1) was used to assess the mature RNA. The TaqMan endogenous control (*GAPDH*; Hs99999905_m1) was used to normalize mRNA expression. Comparative real-time PCR was performed in triplicate. The 2^−ΔΔCt^ method was used to calculate the relative *MET* expression. 

### 2.16. Counting Exosome Particles

The Izon qNano system using TRPS technology (Izon Science, Christchurch, NZ, USA) was utilized to measure the size and number of particles isolated from the culture media, as previously reported [16]. 

### 2.17. cDNA Microarray Analysis

RNA was collected from mouse OC tissues treated with Met-siExosomes or control exosomes. The NanoDrop One spectrophotometer (Thermo Fisher Scientific) and the Agilent Bioanalyzer (Agilent Technologies, Santa Clara, CA, USA) were used to measure the RNA quantity and quality, as previously described [16]. The Agilent SureScan Microarray Scanner G4900DA (Agilent Technologies) was used to perform a cDNA microarray analysis. The complete microarray dataset has been deposited in the NCBI Gene Expression Omnibus (https://www.ncbi.nlm.nih.gov/geo/, accessed on 20 October 2020) and designated as GSE159573. A one-way analysis of variance (ANOVA) was applied to perform a gene expression analysis to identify differentially expressed genes. The *p*-values and fold-changes were calculated for each analysis.

### 2.18. Animal Experiments

Female anthemic BALB/c nude mice (4–6 weeks old) were procured from CLEA (Tokyo, Japan). The Institutional Animal Care and Use Committee of Osaka University approved all the animal experiments (No. J006461-010). One million ES-2 or SKOV3-13 cells were intraperitoneally injected. For the ex vivo imaging, IVIS Limina II (PerkinElmer, Shelton, CT, USA) was used to image the accumulation of siExosomes, which were pre-labeled with the lipophilic fluorescent tracer DiR (#125964, Summit Pharmaceuticals International Corporation, Tokyo, Japan).

### 2.19. Statistical Analysis

A statistical analysis was performed using the JMP^®^Pro software, version 15.1.0 (SAS Institute Japan Ltd., Tokyo, Japan). All the data were normally distributed. The F-test was used to evaluate equal variances. The data are expressed as the mean ± standard error of the mean. A one-way ANOVA and the Bonferroni correction for multiple comparisons were used to analyze the differences. Differences were considered statistically significant at a *p*-value of <0.05. 

## 3. Results

### 3.1. MET Is Highly Expressed in OC and Its Expression Is Associated with a Poor Prognosis in Patients with OC

**Figure 1:**
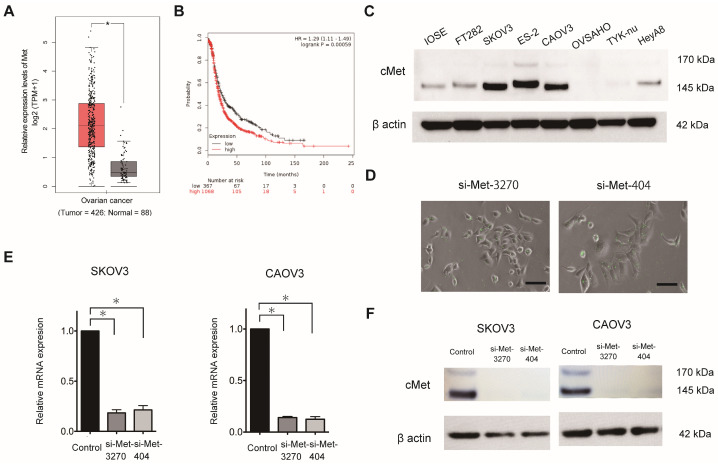
Therapeutic potential of the *MET* oncogene for ovarian cancer (OC). (**A**) The relative expression of *MET* in OC tissues (*n* = 426) and normal ovarian tissues (*n* = 88) was evaluated using GEPIA. Red and black boxes represent the relative expression of *MET* in OC and normal tissues, respectively. * *p* < 0.05. (**B**) Kaplan–Meier plotter (http://kmplot.com/analysis/, accessed on 14 March 2023) analysis of the progression-free survival (PFS) data of patients with OC (*n* = 1435). Black line: low-MET-expression group; and red line: high-*MET*-expression group. (**C**) Western blotting. c-Met expression in OC cell lines. Immortalized ovarian surface epithelium and FT-282 were used as benign controls. (**D**) Representative fluorescence microscopy images. FAM-labeled Met siRNAs (si-Met-3270 and si-Met-404) were transfected into SKOV3 cells. Green, fluorescent spots are shown in the cells. Scale bar: 20 µm. (**E**) Results of qRT-PCR analysis to evaluate *MET* expression relative to that of GAPDH, as calculated using the 2^−ΔΔCt^ method. Relative fold-differences are presented. Results are shown as the mean ± standard error of the mean. SKOV3, *n* = 3; CaOV3, *n* = 3. (**F**) Western blotting. Met siRNAs (si-Met-3270 and si-Met-404) inhibited c-Met expression in SKOV3 (**left**) and CaOV3 (**right**) cells. File S1 in Supplementary Materials: Original Western blot images. The data were analyzed using one way ANOVA. * *p* < 0.05.

Previous studies, including ours [22], have revealed that the *MET* oncogene encoding c-Met plays a pivotal role in OC progression and may represent a promising treatment target. Therefore, in this study, we silenced *MET* expression using siRNAs against this oncogene. GEPIA revealed that *MET* mRNA was more significantly and highly expressed in OC tissues than in normal tissues (Figure 1A). Furthermore, a public database including 1435 patients with OC (Kaplan–Meier plotter) revealed that patients with a higher *MET* mRNA expression exhibited a significantly shorter progression-free survival (PFS) rate than those with a lower expression (Figure 1B). In addition, Western blotting confirmed c-Met expression in various OC cell lines (Figure 1C), and four of six cell lines, namely, SKOV3, ES2, CaOV3, and HeyA8, exhibited high c-Met expression compared to immortalized ovarian surface epithelium and FT-282 cells (generously gifted by Dr. Ronny Drapkin (University of Pennsylvania)), that is hTERT-immortalized cells isolated from the fallopian tube of a female donor.

### 3.2. Synthesis of siExosomes

Here, we aimed to establish a novel siRNA therapy using exosomes as carriers. To identify the candidate siRNAs that can efficiently suppress c-Met expression in OC, we used two FAM-labeled siRNAs that were synthesized as previously described and named them si-Met-3270 and si-Met-404. These siRNAs were successfully transfected into the OC cell lines SKOV-3 and CaOV-3, as confirmed via fluorescence microscopy (Figure 1D). *MET* expression was inhibited at the mRNA (Figure 1E) and protein (Figure 1F) levels. Therefore, we selected si-Met-3270 and si-Met-404 as the candidates for siRNA replacement exosomal therapy.

### 3.3. Fibroblast-Derived Exosomes Can Serve as a Carrier for siRNA Replacement Exosomal Therapy

**Figure 2:**
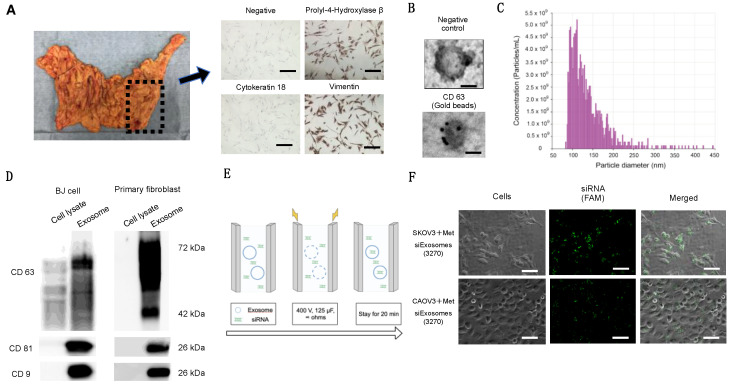
Synthesis of Met-siExosomes. (**A**) Left, the omentum collected from a patient who underwent a primary debulking surgery for ovarian cancer (OC). Right, representative immunohistochemical staining of the patient-derived fibroblast. Fibroblasts exhibited negative staining for cytokeratin 18 but positive staining for poly-4-hydroxylase β and vimentin (scale bar: 100 µm). (**B**) Transmission electron micrographs of the purified exosomes secreted from patient-derived fibroblasts. Scale bar: 100 nm. Upper, negative control; and lower, stained with CD63-targeting gold beads. (**C**) Concentration and size distribution of the nanosized particles in the exosome suspension, as measured using the qNano system. (**D**) Western blotting results of the exosomes and whole-cell lysates collected from BJ cells (**left**) and patient-derived exosomes (**right**). (**E**) Schematic illustration of the protocol for entrapping *MET* siRNAs into exosomes via electroporation. (**F**) Representative fluorescence microscopy images. OC cells (SKOV3 and CAOV3) were treated with Met-siExosomes (3270). *MET* siRNAs were labeled with FAM. Upper, SKOV3 plus Met-siExosomes; and lower, CAOV3 plus Met-siExosomes. The white bar represents 100 µm.

The omentum was obtained during gynecological surgery, and the fibroblasts were isolated and cultured from the omentum (Figure 2A). The fibroblasts exhibited a fusiform morphology. Immunohistochemistry revealed positive staining for vimentin and propel 4-hydroxylase, a fibroblast marker, and negative staining for cytokeratin-18 (Figure 2B). The exosomes were isolated from the supernatants of patient-derived fibroblasts and the human foreskin fibroblast cell line (BJ) via ultracentrifugation, as described previously [16]. Electron microscopy revealed that the collected particles had a round morphology (Figure 2B). Furthermore, a nanoparticle analysis revealed that the particle size was approximately 100–150 nm (Figure 2C). Western blotting revealed that these exosomes expressed CD9, CD63, and CD81, all of which are representative exosome markers; these findings indicate that the collected microparticles were predominantly exosomes (Figure 2D). Next, we incorporated the FAM-labeled siRNAs into the exosomes using electroporation (Figure 2E) to develop Met-siExosomes. OC cells (SKOV3 and CAOV3) were treated with Met-siExosomes. Fluorescence microscopy detected green, fluorescent siRNA in OC cells (Figure 2F).

### 3.4. OC Cells Can Successfully Uptake Met-siExosomes, and Exosomes Can Inhibit c-Met Expression and OC Cell Proliferation, Migration, and Invasion

**Figure 3:**
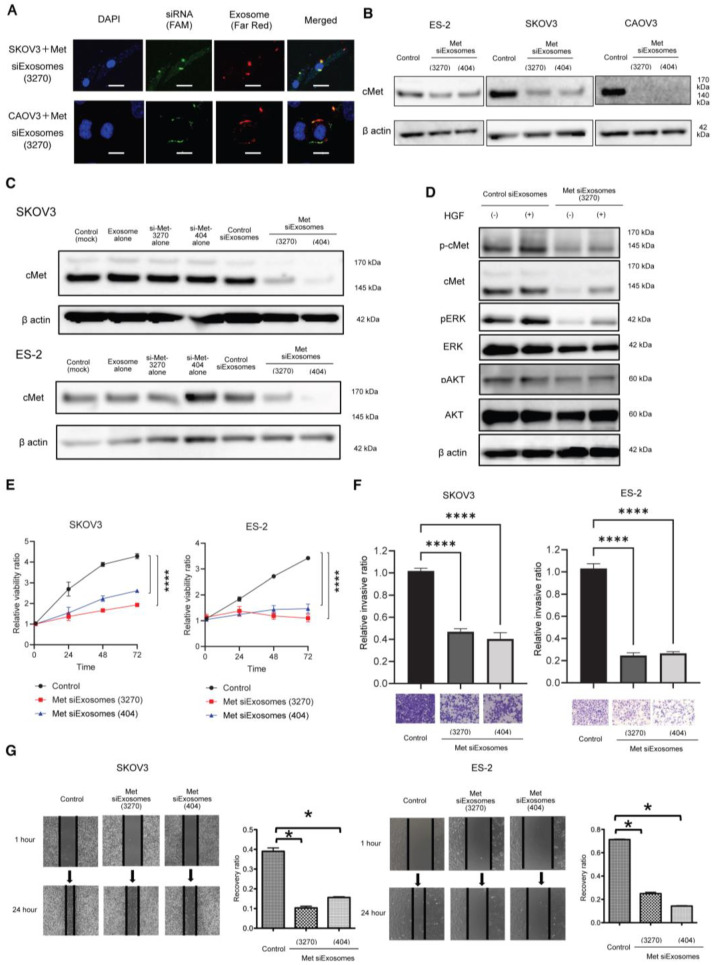
Efficacy of Met-siExosomes in ovarian cancer (OC) cells in vitro. (**A**) Representative confocal microscopy images. OC cell lines (upper, SKOV3; and lower, CaOV3) were cultured for 12 h in the presence of CellVue Claret-labeled Met-siExosomes (3240). Blue: nuclei were counterstained with DAPI; far-red: CellVue Claret (exosomes); green: FAM-labeled siRNAs. Scale bar: 20 µm. (**B**) Western blotting. Met-siExosomes (3270 and 404) successfully inhibited c-Met expression in three OC cell lines (left, ES-2; middle, SKOV3; and right, CaOV3). (**C**) Western blotting. Met-siExosomes (3270 and 404) successfully inhibited c-Met expression in OC cell lines (upper, SKOV3; and lower, CaOV3), whereas exosomes or *MET* siRNA alone did not affect c-Met expression. (**D**) Western blotting. SKOV3 cells were treated with Met-siExosomes (3270) and the corresponding control siRNA-loaded exosomes (control siExosomes). Cell lysates were collected before and after HGF stimulation (40 ng/mL, 10 min). (**E**) In vitro cell proliferation assay. OC cells (5 × 10^3^ cells) were plated onto 96-well plates and treated with control siExosomes and Met-siExosomes (3270 and 404) (left, SKOV3; and right, ES-2). Thereafter, they were incubated for 72 h. (**F**) In vitro Matrigel invasion assay. OC cells (left, SKOV3; and right, ES-2) were pretreated with Met-siExosomes or control siExosomes for 24 h, plated onto a Matrigel-coated 24-well chamber, and allowed to invade for 24 h. The number of invading cells was counted; the relative number of invading cells is presented. (**G**) Wound-healing assay. Cells (left, SKOV3; and right, ES-2) were pretreated with Met-siExosomes or control siExosomes for 24 h and plated on a six-well chamber until confluency. Then, they were scratched and allowed to migrate for 24 h. All the experiments were performed in triplicate. The results in the graphs are represented as the mean ± standard error of the mean. The data were analyzed using Student’s *t*-test. * *p* < 0.05 and **** *p* < 0.0001.

To further verify the uptake of exosomes into OC cells, the exosomes were fluorescently labeled with far-red dye before electroporation with green, fluorescent siRNA. Then, the OC cells were treated with Met-siExosomes. Confocal microscopy revealed that the green, fluorescent siRNAs were taken up by the OC cells and co-localized with red fluorescent-labeled exosomes in SKOV3 and ES-2 cells (Figure 3A). Met-siExosomes were constructed using two siRNA sets (3270 and 404); these Met-siExosomes successfully inhibited c-Met expression in ES-2, SKOV3, and CAOV-3 cells (Figure 3B). Although c-Met expression was not affected by the treatment with exosomes or siRNAs alone in SKOV3 and ES-2 cells, Met-siExosomes inhibited c-Met expression in these cells (Figure 3C). These data suggest that patient-derived exosomes can function as an siRNA carrier. HGF/scatter factor (SF), a ligand for c-Met receptor tyrosine kinase which binds to the c-Met protein, phosphorylates the c-Met protein and activates several signaling pathways, including the ras/mitogen-activated protein kinase and phosphatidylinositol 3-kinase/AKT signaling pathways [20]. Therefore, we examined whether Met-siExosomes could inhibit the HGF/c-Met signaling pathway. Compared with the control siExosomes, the pretreatment with Met-siExosomes suppressed HGF/SF stimulation-induced c-Met phosphorylation and inhibited the phosphorylation of ERK and AKT (Figure 3D). A modified MTS assay revealed that the Met-siExosomes significantly inhibited OC cell proliferation (SKOV3 and ES-2 cells) while the exosomes alone did not affect cell proliferation (Figure 3E). Furthermore, the in vitro invasion assay revealed that the Met-siExosomes inhibited OC cell invasion (Figure 3F). Finally, the wound-healing assay revealed that the siExosomes significantly suppressed OC cell migration (Figure 3G). Collectively, Met-siExosomes can inhibit the proliferation, migration, and invasion of OC cells in vitro.

### 3.5. siExosomes Are Specifically Accumulated in Tumors in a Xenograft Mouse Model

**Figure 4:**
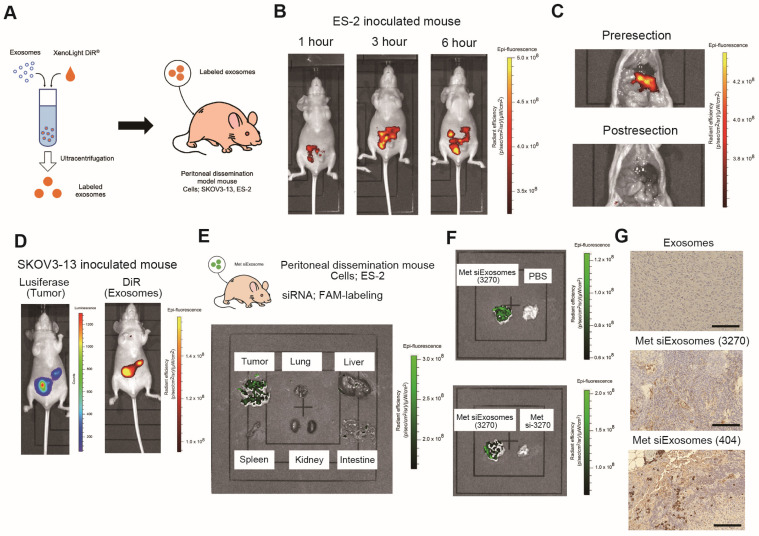
Biodistribution of Met-siExosomes in mice. (**A**) Schematic illustration of the protocol for detecting exosomes in vivo. (**B**) Representative images of the accumulation of DiR-labeled exosomes in mice. Twenty-one days after the inoculation of ES-2 cells, mice were intraperitoneally treated with XenoLight DiR-labeled exosomes, followed by a biodistribution evaluation. Images were captured using an IVIS imaging system 1, 3, and 6 h after intraperitoneal injection. (**C**) Representative images of exosome accumulation in the omental tumors. Upper, pre resection of the omentum; and lower, post resection of the omentum. (**D**) Representative images of a mouse 28 days after inoculating SKOV3-13-luci cells treated with DiR-labeled exosomes. Left, luciferase activities in the tumors; and right, siExosome accumulation. Images were simultaneously captured using an IVIS imaging system. (**E**) Imaging of the indicated organs for detecting siExosomes. FAM-labeled exosomes were intraperitoneally injected into ES-2-bearing mice. After 24 h, the images of the indicated organs were captured. (**F**) Comparison of the inoculated ES-2 tumors. Upper, Met-siExosomes and PBS; and lower, Met-siExosomes and FAM-labeled siRNA alone. (**G**) Representative immunohistochemical staining of the tumors harvested from the mice treated with FAM-labeled siRNAs alone and Met-siExosomes (3270 and 404). Tumors were stained with anti-FAM antibodies. The bar represents 100 µm.

Before investigating the therapeutic potential of the siExosomes, their in vivo biodistribution was confirmed in BALB/c nu/nu mice inoculated with OC cells. ES-2 cells were inoculated into female mice. One week after inoculation, DiR-labeled exosomes or PBS were intraperitoneally injected into the mice, followed by ex vivo imaging (Figure 4A). Mice were observed 1, 3, and 6 h after intraperitoneal injection; the DiR-labeled exosomes gradually accumulated at specific intraperitoneal lesion sites (Figure 4B). Six hours after injection, the mice were euthanized, and IVIS imaging was performed under a laparotomy. The DiR-labeled exosomes strongly accumulated in the mouse omentum, including the major peritoneal dissemination sites of ES-2 cells. Indeed, the fluorescence signals almost completely disappeared after intraperitoneal tumor resection (Figure 4C). Similar experiments were performed using SKOV3-13 cells stably transfected with firefly luciferase. DIR-labeled exosomes were injected into SKOV3-13-inoculated mice, followed by IVIS imaging after 6 h. The DiR-accumulated sites almost corresponded with the luciferase activity sites (Figure 4D), suggesting the accumulation of DiR-labeled exosomes in the tumors. Next, to further confirm whether the targeting siRNAs were successfully delivered to the OC cells, ES-2-inoculated mice were administered Met-siExosomes, PBS, or siRNA alone every 2 days. The mice were euthanized 2 weeks after inoculation, followed by harvesting of the inoculated tumors. IVIS and immunohistochemistry were performed to detect FAM-labeled siRNAs in vivo. Ex vivo imaging revealed the accumulation of FAM-labeled siRNAs only in the tumors of mice that received Met-siExosomes but not in other organs such as the liver and the spleen (Figure 4E); in contrast, non-fluorescent signals were observed in the tumors of mice that received PBS and FAM-labeled siRNAs alone (Figure 4F). Furthermore, these tumors were immunostained with FAM antibodies to identify the presence of FAM-labeled siRNAs. Strong FAM staining was only observed in the tumors of mice that received Met-siExosomes (Figure 4G). Collectively, these results suggest that the intraperitoneal injection of Met-siExosomes into mice results in their predominant accumulation in inoculated tumors, with the successful delivery of encapsulated siRNAs. These findings indicate that patient-derived exosomes can function as a carrier to deliver siRNAs to peritoneally disseminated tumors in vivo.

### 3.6. Met-siExosomes Inhibit the Peritoneal Dissemination of OC in the Xenograft Mouse Model

**Figure 5:**
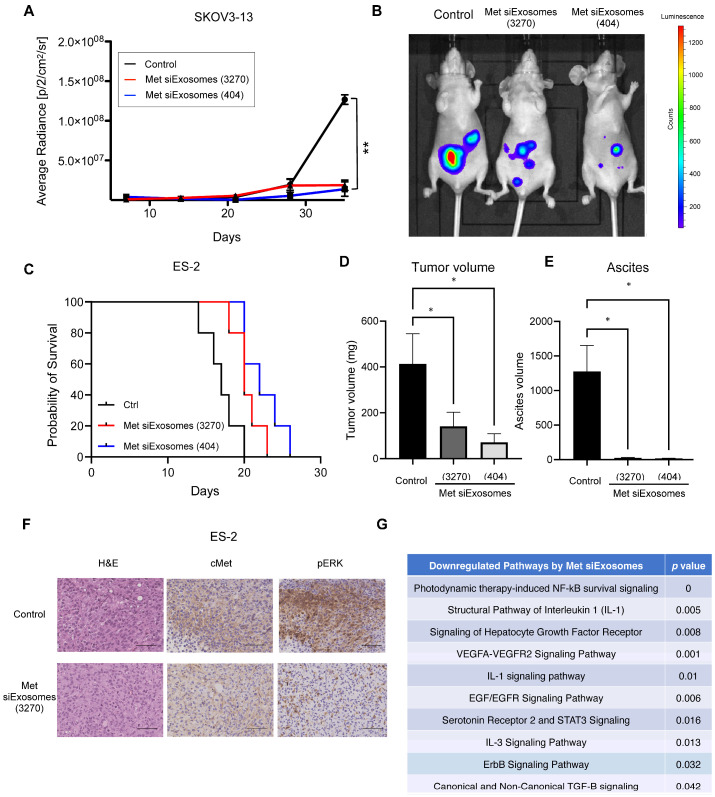
Met-siExosomes inhibit the peritoneal dissemination of ovarian cancer (OC) in mice by suppressing HGF/Met signaling. (**A**) The luciferase activities of the peritoneal tumors were measured each week after injecting SKOV3-13 cells. Treatment with Met-siExosomes (3270, red; and 404, blue) was initiated 1 week after the inoculation. Control siExosome was used as the control (black). *Y*-axis, radiance (p/s/cm^2^/sr), *n* = 5. (**B**) Representative images of the mice inoculated with SKOV3-13 after treatment with control siExosomes and Met-siExosomes (3270 and 404). (**C**) Kaplan–Meier survival curve of ES-2 tumor-bearing mice (*n* = 5 each for control siExosomes, Met-siExosomes (3270), and Met-siExosomes (404)). (**D**,**E**) One million ES-2 cells were injected into female BALB/c mice. Three days after the injection, treatment with two sets of Met-siExosomes (3270 and 404) and control siExosomes was initiated every 48 h. Three weeks after the injection, the total tumor burden (**D**) and ascites volumes (**E**) in the mice were measured. (**F**) Representative images of the inoculated ES-2 tumors in mice. H&E staining, c-Met, and p-ERK expression in a serial section of the xenograft tumors. (**G**) Summary of pathway analysis via gene expression microarray comparing the xenograft ES-2 tumors of mice treated with control siExosomes and Met-siExosomes. The pathways downregulated by Met-siExosomes are listed. Results are expressed as the means ± standard error of the mean. * *p* < 0.05, and ** *p* < 0.01.

Finally, the therapeutic effects of Met-siExosomes were evaluated. One million SKOV3-13 cells were inoculated into female BALB/c mice. The exosome source was patient-derived fibroblasts. Two weeks after inoculation, the mice were repeatedly injected intraperitoneally with two sets of Met-siExosomes (3270 and 404) every 48 h. Scrambled siRNA was used as the control (control siExosomes). Luciferase activity was measured every week to assess the treatment efficacy of the siExosomes. Five weeks after the intraperitoneal injection of the siExosomes, luciferase activity significantly decreased, reflecting the tumor volume in the peritoneal cavity of the Met-siExosome-treated mouse group compared with that treated with control siExosomes (*p* < 0.01) (Figure 5A,B). Similar in vivo experiments were performed using ES-2 cells. One million ES-2 cells were inoculated into female BALB/c mice. Three days after injection, the mice were treated with two sets of Met-siExosomes (3270 and 404), as described above. The median overall survival of the mice that received these Met-siExosomes significantly increased compared with those that received control siExosomes (control, 17.0 ± 2.2 days vs. siExosome (3270) exosomes, 20.4 ± 1.8 days vs. siExosome (404), 22.4 ± 2.6 days, *p* = 0.0035,) (Figure 5C). In another set of experiments using ES-2, 3 weeks after injection (18 days after treatment), the tumor burden and the amount of ascites were examined between the groups (Figure 5D,E). The tumor burden and ascites formation were significantly lower in the Met-siExosome groups than in the control siExosome group (tumor weight: control siExosomes, 413.1 ± 66.0 mg; Met-siExosomes (3270), 141.1 ± 30.7 mg; and Met-siExosomes (404), 71.6 ± 17.5 mg, *p* < 0.01; and amount of ascites: control siExosomes, 1275.0 ± 188.7 mL; Met-siExosomes (3270), 25.0 ± 3.5 mL; and Met-siExosomes (404), 17.5 ± 1.4 mL, *p* < 0.01); this suggests the treatment efficacy of siExosomes in vivo. Furthermore, the inoculated tumors were harvested, and the efficacy of the Met-siExosomes in vivo was assessed using immunohistochemistry and a cDNA microarray analysis. The immunohistochemical analysis revealed that the Met-siExosomes (3270) inhibited c-Met expression and downstream molecules such as p-ERK compared with the control siExosomes in ES-2-inoculated mice (Figure 5F). Using a cDNA microarray, a pathway analysis was performed using the PathVisio software version 3.3.0. The data demonstrated that cancer growth, proliferation, and HGF-related pathways were significantly suppressed in the Met-siExosome group compared to the control group (Figure 5G). Collectively, the intraperitoneal injection of the Met-siExosomes successfully suppressed c-Met expression and inhibited tumor and ascites formation, thereby increasing the survival time of the mice. This indicates that exosomes can serve as an ideal carrier for siRNA delivery in treating the peritoneal dissemination of OC. 

## 4. Discussion

In this study, exosomes were collected from patient-derived fibroblasts and used as a carrier of tumor suppressor siRNAs. Encapsulated siRNAs were specifically delivered to the intraperitoneal tumors of the OC mouse model: the siRNAs inhibited the peritoneal dissemination of OC cells and prolonged mouse prognosis. Thus, we present the potential of exosomes as a future carrier of nucleic acid compounds to cure OC. 

Despite the development of novel molecular targeted therapies, such as PARP inhibitors, the prognosis of OC remains poor, particularly in advanced cases. OC is a highly heterogeneous disease that often leads to extensive peritoneal dissemination and carcinomatous peritonitis, making it challenging to control intra-abdominal lesions. To overcome the challenges associated with achieving a complete cure, the development of personalized treatment regimens for OC has garnered attention [23]. 

Since the demonstration of the robust and specific efficacies of siRNAs in mammalian cells, the potential application of siRNAs in cancer treatment has garnered considerable attention [24]. However, siRNA technology faces multiple challenges with respect to its efficient delivery and instability in vivo. To overcome these shortcomings, various delivery methods, including electroporation, hydrodynamic vein injection, and the synthesis of non-viral vehicles (e.g., polymers, cationic lipids, and nanoparticles), and different chemical modifications of siRNAs have been attempted in vivo [24]. Although some of these approaches have demonstrated successful efficacy without apparent toxicity in preclinical settings, to the best of our knowledge, none have demonstrated efficacies significant enough to go through phase III clinical studies. Hattab D et al. reviewed clinical advances of siRNA-based nanotherapeutics for cancer treatment and described that nine clinical trials have been conducted, including five phase I and four phase II siRNA-based therapies to date [7]. Among those, five clinical trials have already been terminated or completed, three are still ongoing, and one has not yet recruited patients. A phase I study is evaluating the suitable dose and adverse events of KrasG12D siRNA-loaded mesenchymal stromal cells-derived exosomes for patients with metastatic pancreatic cancer with a KrasG12D mutation (NCT03608631; https://clinicaltrials.gov/study/NCT03608631, accessed on 14 March 2024). This study is now recruiting. Recently, zilebesiran, a subcutaneously administered siRNA-targeting liver-expressed angiotensinogen, was developed to treat hypertension [25]. Although siRNAs have opened novel avenues for innovative therapies for many diseases, further exploring siRNA drugs beyond the liver is vital, emphasizing the need for appropriate delivery strategies [26]. Emerging evidence has revealed that exosomes are safe and effective drug carriers in cancer therapy. Compared with liposomes and nanoparticles, exosomes offer a high biocompatibility, a low immunogenicity, the ability to easily penetrate biological barriers, and nontoxic accumulation [27]. Furthermore, chemotherapeutic drugs, tumor-targeting RNAs, or proteins can be easily encapsulated into exosomes by mixing followed by electroporation or sonication [28]. Therefore, the development of drug delivery systems using exosomes is of great scientific significance, with potential clinical applications. 

Here, we chose *MET*, a tyrosine kinase receptor of HGF, as a treatment target because various cancers, including OC, are characterized by an alternating *MET* expression, which is generally associated with a poor prognosis and aggressive phenotypes [29]. Considering that oncogenic *MET* amplification and the activation of downstream effectors such as AKT, ERK, and MEK are sufficient drivers of OC [30], controlling the HGF/MET axis is an attractive and promising target. To date, although various *MET* kinase-specific small-molecule inhibitors and many HGF/MET-neutralizing antibodies have been developed, concomitant therapeutic resistance has interfered with the translation of such candidate drugs into clinical applications [29]. Therefore, the mere inhibition of the HGF/MET axis with a single drug may be insufficient to achieve a treatment effect in clinical settings. 

Drastic improvements in delivery efficacy and selectivity are indispensable, and using exosomes as a carrier may provide this opportunity. In this study, we utilized patient-derived omental fibroblasts as the source of the exosomes. Omentectomy is a common procedure for patients with OC that provides readily accessible omental tissue from which fibroblasts can be isolated. In our experience, from 100 g of omental tissue, approximately 1 × 10^7^ of fibroblasts could be collected and passaged up to five times. During passage, approximately 100 µg of exosomes are obtained from these fibroblasts. Thus, we consider these secreted exosomes to be in ample quantities, suitable for siRNA encapsulation, thereby enabling their use as a novel treatment modality. Our in vitro results demonstrated that these siRNA-loaded exosomes are effectively taken up by cancer cells, leading to the silencing of the target gene. Furthermore, our in vivo experiments revealed that these patient-derived exosomes are efficiently accumulated at tumor sites and significantly inhibit tumor growth, thus offering a promising direction for OC treatment strategies.

The most innovative part of this study is that patient-derived exosomes can be collected from fibroblasts and that these fibroblasts can be easily obtained from the omentum. However, the safety and viability of fibroblast-derived exosomes for human disease are still controversial. Recently, normal skin fibroblast-derived exosomes have been shown to aid in wound healing through boosting immune responses [31,32]. Bhandari R et al. reported that the exposure of dermal fibroblast-derived exosomes to macrophages upregulated the surface expression of CD163, CD206, MHC Class II, and CD16, followed by the increased secretion of IL-6, IL-10, IL-12p40, and TNF. However, cancer cells often program fibroblasts into cancer-associated fibroblasts (CAFs), and a variety of studies have shown that CAF-derived exosomes play pivotal roles in tumorigenesis, tumor cell proliferation, metastasis, drug resistance, and the immune response [33,34]. For instance, Li et al. reported that CAF-derived exosomal TGF-β1 induce epithelial–mesenchymal transition (EMT) and thereby promote the progression and metastasis of ovarian cancer [35]. Thus, fibroblast-derived exosomes might have a detrimental effect on cancer progression. In this study, we used omentum without gross abnormal appearances from patients with stage I ovarian cancer. We have previously reported whether these fibroblast-derived exosomes affect cancer cell behavior [16] and showed that the pathways related to cancer growth and proliferation are not significantly altered by a treatment with fibroblast-derived exosomes [GSE147610; https://www.ncbi.nlm.nih.gov/geo/query/acc.cgi?acc=GSE147610, accessed on 14 March 2024]. Therefore, for the clinical application of this treatment, it would be crucial to collect normal fibroblasts from the omentum, although it might sometimes be difficult to collect normal omentum in patients with multiple omental metastases.

We chose to intraperitoneally administer the encapsulated exosomes. The advantage of intraperitoneal administration is the delivery of a high concentration of the therapeutic agent to the tumor sites. Recent findings suggest that micropinocytosis, a type of endocytosis involving the nonspecific uptake of extracellular materials such as exosomes, is enhanced in cancer cells [36]. The immunostaining analyses revealed that FAM-labeled siRNAs selectively penetrated the tumor, suggesting that siRNA-encapsulated exosomes were more selectively incorporated into the tumor. Therefore, the intraperitoneal administration of encapsulated exosomes may be beneficial in treating peritoneally disseminated OC. To accomplish the clinical application of this strategy, the placement of an intraperitoneal (IP) port into the abdomen would be needed. While there has been clinical evidence of the benefit of postoperative IP chemotherapy in advanced ovarian cancer, it has not been widely adopted, mainly due to its high morbidity, high cost, and logistical difficulties [37]. Recently, Aronson SL et al. showed that the addition of hyperthermic intraperitoneal chemotherapy (HIPEC) to interval cytoreductive surgery resulted in improved progression-free and overall survival rates compared with cytoreductive surgery alone in patients with stage III epithelial ovarian cancer in a phase III trial [38]. Thus, the placement of an IP port into patients’ abdomens during cytoreductive surgery might be a future viable option for the treatment of advanced ovarian cancer. In such a case, the placed port can be used for the IP administration of the engineered exosomes for the cure of patients with intraperitoneally relapsed ovarian cancer who no longer respond to conventional treatments.

The most important requirement for the establishment of exosome-based delivery strategies for cancer treatment is the development of a method of collecting ample volumes of exosomes from patients. Therefore, one of the strengths of this study is that we revealed the possibility of collecting ample volumes of exosomes from patient-derived tissues, which is clinically meaningful. Furthermore, omentum contains fibroblasts as well as adipose cells and macrophages [39], indicating that a variety of exosome types may be obtained. Moreover, considering the less-invasive nature of this surgery, the establishment of exosome-based delivery strategies using patient-derived omentum could lead to the application of laparoscopic omentectomy for OC as well as other types of cancers, such as colorectal cancer or gastric cancer. 

This study has several limitations. First, exosomes were collected using differential ultracentrifugation, which is a complicated method which cannot remove the debris inevitably produced during the collection process. Thus, for applications in clinical settings, easier methods of collecting high-quality exosomes are warranted, such as size exclusion chromatography, dense gradient centrifugation, and immunoaffinity capture [14]. Second, although the omentum was used as the exosome source, a laparotomy must be performed to utilize the omentum. However, in many cases of recurrent OS, surgery is not a treatment option. Third, at present, it is not known how much volume of exosomes can be safely tolerated by patients who receive IP exosome administration. As described above, a phase I study of mesenchymal stromal cells-derived exosomes with KrasG12D siRNA for patients with metastatic pancreas cancer is recruiting (NCT03608631). The primary objectives of this study are to identify the maximum tolerated dose and the dose-limiting toxicities of siRNA-loaded exosomes (NCT03608631; https://clinicaltrials.gov/study/NCT03608631, accessed on 14 March 2024). By accumulating such a clinical trial, we will be able to estimate the tolerable dosage and the safety of Met-siExosomes for patients. Fourth, because the median PFS of patients with advanced OC is 16–21 months [40], it is impractical to store exosomes collected from the omentum during primary surgery to prevent a future recurrence. Therefore, an ideal exosome source is desirable for clinical applications. A recent review revealed that exosomes can be isolated from the immune cells present in patients’ blood, including dendritic cells, natural killer cells, and macrophages [41]. However, ample amounts of exosomes are difficult to collect from immune cells in the peripheral blood; therefore, future studies should focus on developing innovative methods of collecting exosomes to promote their clinical use.

## 5. Conclusions

siRNA replacement therapy using exosomes from patients exhibits promising effects for treating the peritoneal dissemination of OC. Considering that most patients with OC undergo omentectomy, exosomes can be extracted from omental fibroblasts without difficulty, and engineered exosomes can be used as siRNA carriers for future molecular targeted therapies, ultimately resulting in personalized medicine for patients with OC.

## Data Availability

The data generated in this study are publicly available in GEO at GSE GSE159573, and the other data are available from the corresponding author upon reasonable request.

## Supplementary Materials

The following supporting information can be downloaded at: https://www.mdpi.com/article/10.3390/cancers16081482/s1, File S1: Original Western blot images.
