# Supplementary material for: Patient-Derived Exosomes as siRNA Carriers in Ovarian Cancer Treatment
Source: Cancers (Basel). 2024 Apr 12;16(8):1482. doi: 10.3390/cancers16081482 (PMC11048711; doi:10.3390/cancers16081482)
Supplement: Supplementary file 1 [file cancers-16-01482-s001.zip › cancers-2861056-supplementary.pptx]

## Slide 1
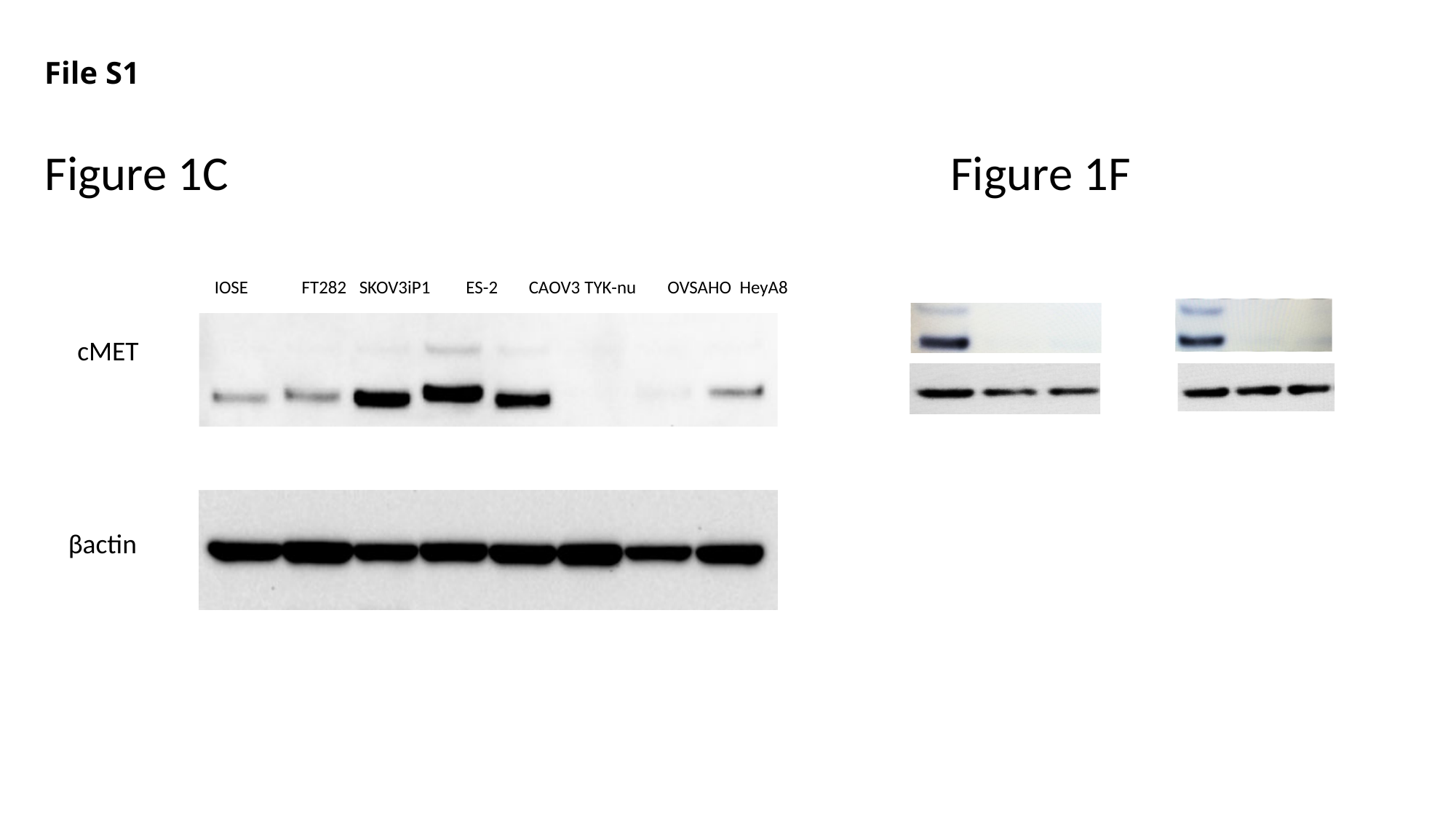

File S1
Figure 1C
Figure 1F
 IOSE　　 FT282 SKOV3iP1　 ES-2 　CAOV3 TYK-nu 　OVSAHO HeyA8
cMET
βactin

## Slide 2
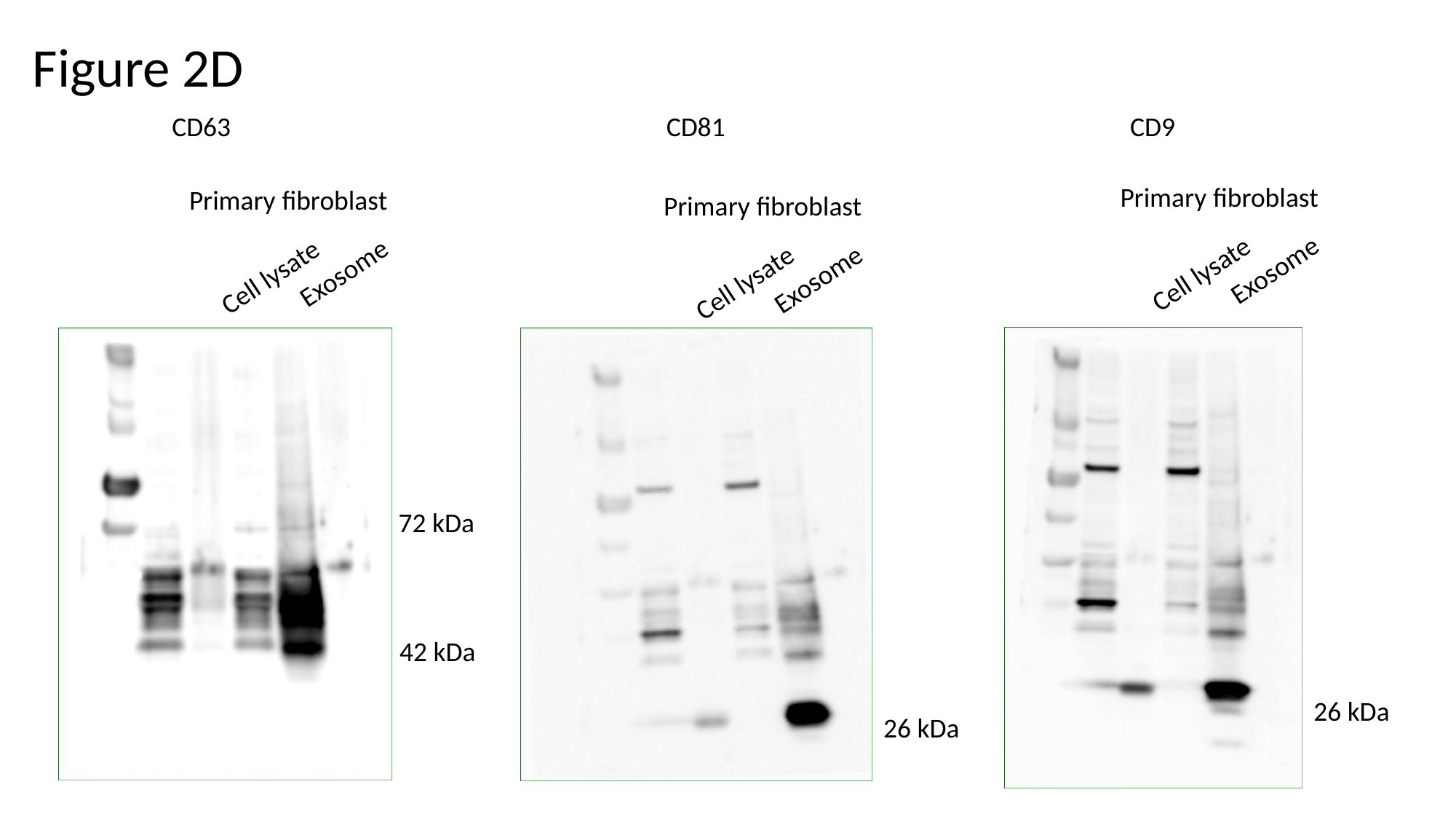

Figure 2D
CD63
CD81
CD9
Primary fibroblast
Primary fibroblast
Primary fibroblast
Exosome
Exosome
Cell lysate
Cell lysate
Exosome
Cell lysate
72 kDa
42 kDa
26 kDa
26 kDa

## Slide 3
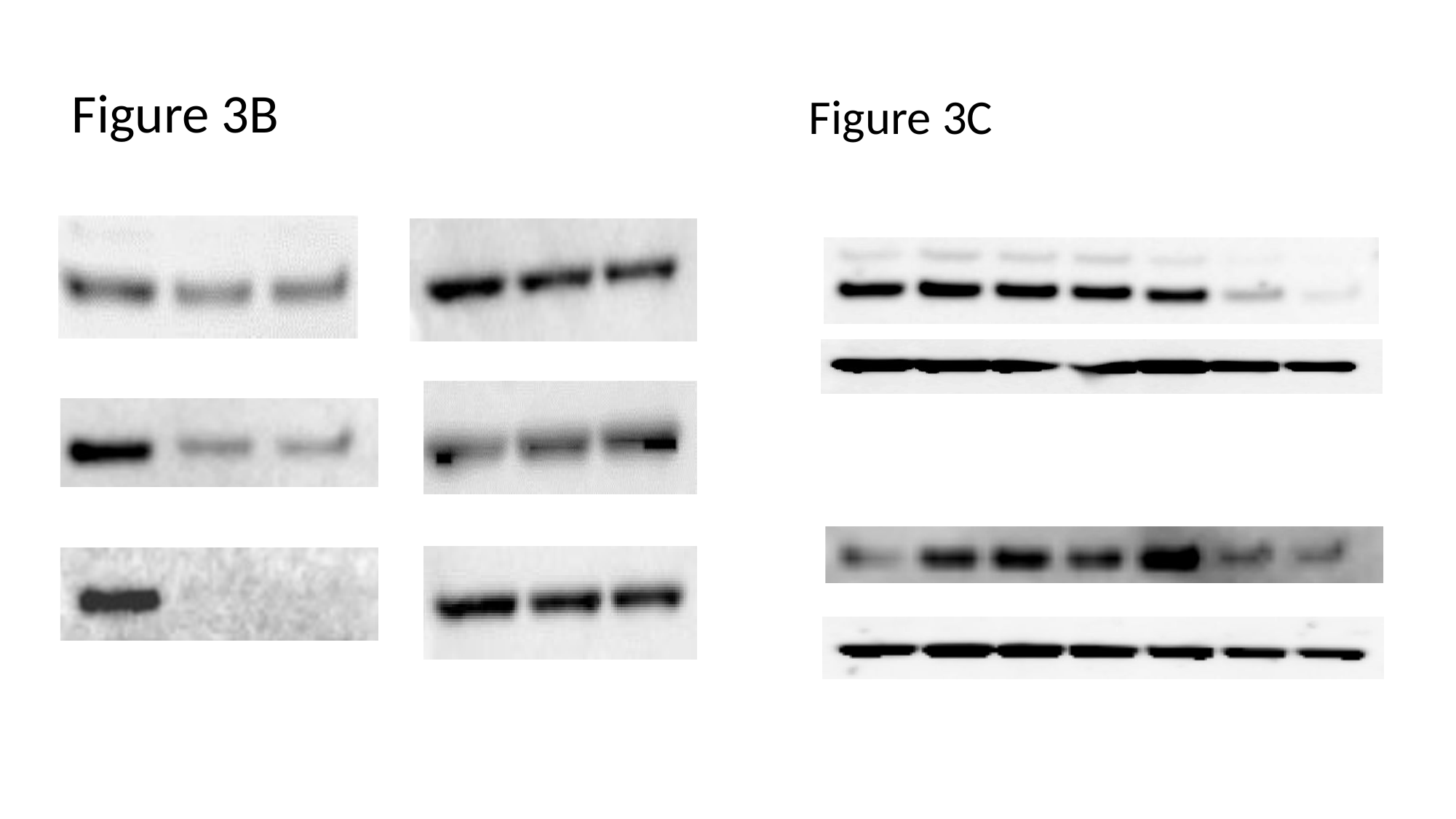

Figure 3B
Figure 3C

## Slide 4
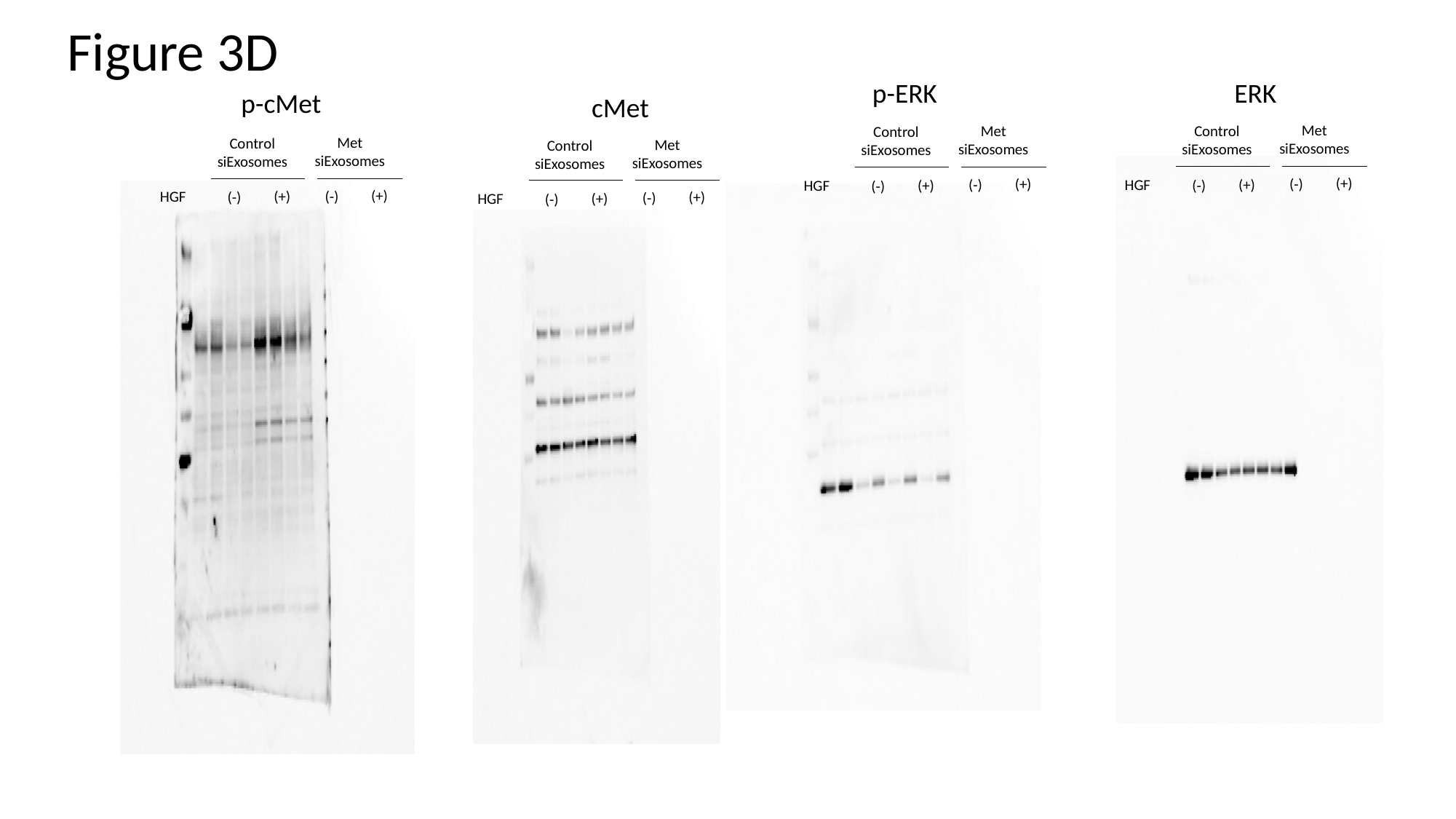

Figure 3D
 p-ERK
 ERK
 p-cMet
 cMet
Met siExosomes
Control siExosomes
Met siExosomes
Control siExosomes
Met siExosomes
Control siExosomes
Met siExosomes
Control siExosomes
(+)
(-)
(+)
(+)
HGF
(-)
(-)
(+)
HGF
(-)
(+)
(-)
(+)
HGF
(+)
(-)
(-)
(+)
HGF
(-)

## Slide 5
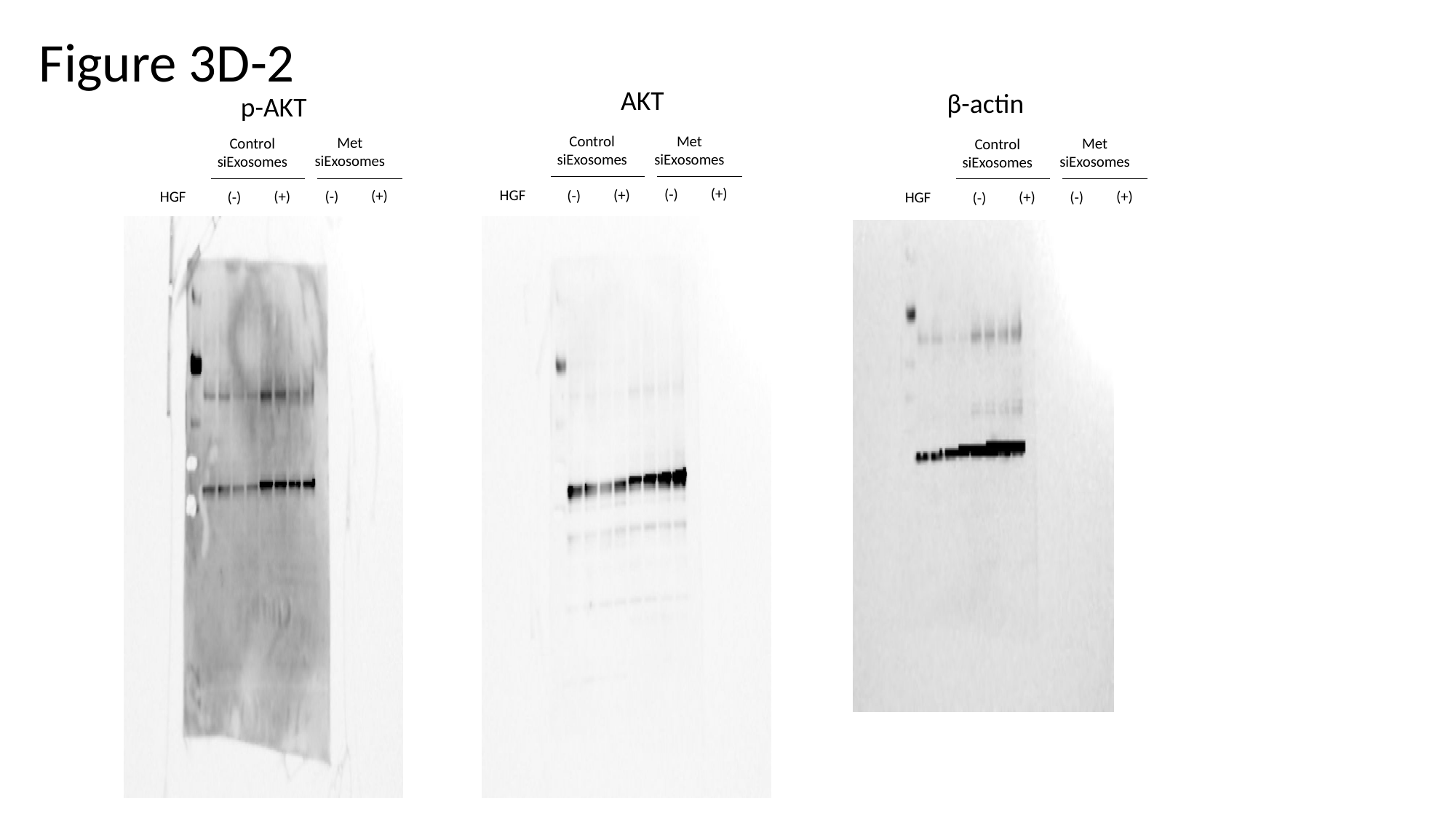

Figure 3D-2
 AKT
 β-actin
 p-AKT
Met siExosomes
Control siExosomes
Met siExosomes
Control siExosomes
Met siExosomes
Control siExosomes
(+)
(-)
(+)
HGF
(+)
(-)
(+)
(-)
(+)
HGF
(-)
(+)
HGF
(-)
(-)
